# Individual and community level factors associated with health facility delivery: A cross sectional multilevel analysis in Bangladesh

**DOI:** 10.1371/journal.pone.0211113

**Published:** 2019-02-13

**Authors:** Tanvir M. Huda, Morseda Chowdhury, Shams El Arifeen, Michael J. Dibley

**Affiliations:** 1 The University of Sydney, Faculty of Medicine and Health, School of Public Health, NSW, Australia; 2 International Centre for Diarrhoeal Disease Research, Bangladesh, Dhaka, Bangladesh; 3 Health Nutrition and Population Programme, BRAC, Dhaka, Bangladesh; Erasmus Medical Center, NETHERLANDS

## Abstract

**Introduction:**

Improving maternal health remains one of the targets of sustainable development goals. A maternal death can occur at any time during pregnancy, but delivery is by far the most dangerous time for both the woman and her baby. Delivery at a health facility can avoid most maternal deaths occurring from preventable obstetric complications. The influence of both individual and community factors is critical to the use of health facility delivery services. In this study, we aim to examine the role of individual and community factors associated with health facility-based delivery in Bangladesh.

**Methods:**

This cross-sectional study used data from the Bangladesh Maternal Mortality Survey. The sample size constitutes of 28,032 women who had delivered within five years preceding the survey. We fitted logistic random effects regression models with the community as a random effect to assess the influence of individual and community level factors on use of health facility delivery services.

**Results:**

Our study observed substantial amount of variation at the community level. About 28.6% of the total variance in health facility delivery could be attributed to the differences across the community. At community level, place of residence (AOR 1.48; 95% CI 1.35–1.64), concentration of poverty (AOR 1.15; 95% CI 1.03–1.28), concentration of use of antenatal care services (AOR 1.11, 95% CI 1.00–1.23), concentration of media exposure (AOR 1.20, 95% CI 1.07–1.34) and concentration of educated women (AOR 1.12, 95% CI 1.02–1.23) were found to be significantly associated with health facility delivery. At individual level, maternal age, educational status of the mother, religion, parity, delivery complications, individual exposure to media, individual access to antenatal care and household socioeconomic status showed strong association with health facility-based delivery.

**Conclusion:**

Our results strongly suggest factors at both Individual, and community level influenced the use of health facility delivery services in Bangladesh. Thus, any future strategy to improve maternal health in Bangladesh must consider community contexts and undertake multi-sectorial approach to address barriers at different levels. At the individual level the programs should also focus on the need of the young mother, the multiparous the less educated and women in the poorest households.

## Introduction

Global maternal mortality ratio has fallen by nearly 44% between 1990 and 2015[1]. Despite the significant reduction over the last two decades, the mortality ratio is still unacceptably high in many low and middle-income countries. In 2015, approximately 5500 women died (95% CI 3900 to 8800) in Bangladesh from maternal causes[2]. The lifetime risk of women to die from maternal causes is estimated to be 1 in 240 in Bangladesh[2]. Improving maternal health thus remains one of the targets of Sustainable Development Goals (SDG). SDG has set a goal of reducing the maternal mortality ratio (MMR) to less than 70 per 100,000 live births by 2030[3].

As the delivery process can result in unexpected complications, health facility delivery or delivery by a skilled attendant is crucial. It is now well established that ensuring skilled attendant at birth, or health facility delivery, can avoid most maternal deaths occurring from preventable obstetric complications and thus can make a critical difference to the survival of the mother[4, 5]. An analysis of secondary data from 48 low and middle-income countries reported that in Sub Saharan Africa, South Asia, and Southeast Asia, more than 70% of all births in the lowest two wealth quintiles occurred at home [6]. In Bangladesh only around 37% of women delivered in a designated health care facility[7]. Critical to improving these rates is an understanding of the multilevel factors associated with utilisation of health facility delivery services.

Several studies conducted in Bangladesh and other similar settings have attempted to identify the determinants of health facility delivery. Most studies have focused on individual and health system factors and demonstrated a significant effect of those factors on the use of health facility delivery services[8–13]. Although studies have reported similar sets of determinants, the effect size differs from one geographic area to another. Therefore, it is possible that unobserved community factors also influenced the location where women deliver. Few studies have already documented the role of community or social factors in the utilisation of maternal health services in South Asia and Africa[14, 15]. However, the role of community factors on the utilisation of health facility delivery services is still less understood in Bangladesh. We, therefore, planned to examine a range of individual and community factors and measure their extent of influence on health facility-based delivery in Bangladesh.

## Methods

### Data sources

We used data from 2010 Bangladesh Maternal Mortality Survey[16], the largest national household survey designed to provide the national estimate of maternal mortality ratio (MMR) and information on family planning, antenatal, delivery, postnatal, and emergency obstetric care.

The sampling frame for the BMMS survey was divided into urban and rural areas. The primary sampling unit (PSU) for the urban and rural areas was the ward and union respectively. For each selected PSU, two mohallas (the next administrative unit for urban area) or a mouza (the next administrative unit for rural area) were randomly selected and segmented into clusters. A cluster was then randomly selected from each selected mohallas or mouza. A total of 2,708 clusters were selected including 1,142 urban and 1,566 rural clusters. From these clusters, 175,600 households (around 65 household from each cluster) were then randomly selected for the survey, of which 168,629 were successfully interviewed. From these 168,629 households, 175,621 women were interviewed for the measurement of maternal mortality. Among them, information on antenatal, delivery and postnatal care were collected from 28,032 women who had a birth in the five years preceding the survey. We have included all 28,032 women in our study.

### Variables

#### Outcome variable

Our outcome variable is whether a mother delivered at home or in a health facility. A birth is categorised as health facility-based if it occurred at a private, public or non-governmental clinic.

#### Explanatory variables

We selected explanatory variables based on Andersen’s health-seeking behavioral model. According to Andersen’s behavioral model, predisposing, enabling, and need factors at the individual and community levels are responsible for increasing health-seeking behavior and health facilities utilization[17–19]. We have included need factors at the individual level while predisposing and enabling factors were included at community level. For community-level factors, we have developed several binary variables by aggregating the individual level characteristics at the cluster level.

#### Individual level factors

Age at birth (less than 20, 20–34 years, 35 years and above)

1. Maternal Education (No education, primary incomplete, primary completed, higher). Primary complete is defined as completing grade 5 and secondary complete is defined as completing grade 10.
2. Religion (Islam, Hinduism and others)
3. Parity (1, 2, 3 and 4 or more),
4. Maternal care seeking practices (at least 3 ANC or any ANC from a medically trained provider that is a qualified doctor, nurse, midwife, paramedic, community skilled birth attendant (CSBA) and others as designated by Govt of Bangladesh[7]),
5. Exposure to mass media (watch TV or read the newspaper at least once in a week)
6. Pregnancy complications in her last pregnancy (convulsion/fits, High BP or Edema, Severe bleeding, mal-presentation and prolonged labour).
7. Household wealth index as a proxy for the women socioeconomic condition.

### Community level factors

1. Region (Sylhet, Barisal, Chittagong, Dhaka, Khulna, Rajshahi)
2. Area of residence (Urban and Rural)
3. High concentration of exposure to mass media in the community (whether or not more than 50% population of the cluster read the newspapers at least once in a week or watch TV at least once in a week),
4. High concentration of use of ANC in the community (whether or not more than 50% pop of the cluster had at least 1 ANC check from a medically trained provider or had at least 3 ANC checks),
5. High concentration of educated women in the community (whether or not more than 50% pop of the cluster had at least eight years of education)
6. High concentration of wealth in the community (whether or not more than 50% of the population are in the top 3 wealth quintiles)

### Statistical analysis

We performed multilevel regression analyses to assess the individual, household and community level factors associated with health facility delivery. We used random effects logistic model (also known as the mixed effect or random intercept model) with two levels to assess the influence of individual and community factors on the use of health facility-based delivery services. Multilevel modelling technique was used to take account the hierarchical structure of our data. In our survey, women were nested within households and households were nested within clusters. We have considered clusters as our random effect to account for the unexplained variability at the community level. All analysis was done on weighted data.

We first constructed an empty” model (model i), which only includes a random intercept. An empty random effect model will provide an estimation of the degree of correlation in the health facility delivery that exists at the community level (cluster). We then included all individual factors in the model (model ii). Finally, we added the community level factors (model iii) to examine which contextual factors have the most influence on the use of a health facility for delivery care. For all models, we presented the odds ratio and associated 95% confidence intervals. We did all statistical analyses using the Stata statistical software, version 15.

### Ethics

The present study relied upon secondary analysis of anonymous, publicly available household survey data from BMMS 2010. The BMMS 2010 survey was approved by the Ethical Review Committee (ERC) of the Bangladesh Medical Research Council (BMRC). All study participants gave informed consent before participation. The raw data of BMMS 2010 is publicly available. We have downloaded the data with permission from the Measure Evaluation.

## Results

We included 28,032 mothers in our analysis. We presented the percentage of women using health facility delivery by the individual, characteristics in Table 1. The overall use of health facility delivery services in our sample was 21.6% (95% CI 20.8%, 22.5%). The results showed significant differences in the use of health facility-based delivery services between catergoreis of maternal age, education, religion, parity, exposure to mass media, household wealth and complications experienced during pregnancy. The utilisation of health facility delivery service was higher among the younger, educated and women from affluent households. The rate was slightly lower among the Muslim women. Also, women who reported three or more ANC visit from any provider had a higher rate of health facility delivery than women who only reported one ANC visit. Similarly, women who reported ANC from a medically trained provider had a higher rate of health facility delivery than women who reported ANC from a non-medically trained provider. Also, women with exposure to mass media had a higher rate of health facility delivery.

**Table 1:** Descriptive analysis of individual characteristics according to place of delivery.

	Health facility Delivery		
	No	Yes	P value
	N (%)	N (%)	
Maternal Age			
<20	2,494 (77.1%)	740 (22.9%)	0.0000
20–34	16,888 (77.5%)	4,892 (22.5%)	
35+	2,720(85.1%)	477(14.9%)	
Parity			
1	5,948(66.5%)	2,993 (33.5%)	0.0000
2	6,438(78.2%)	1,795 (21.8%)	
3	4,246(84.3%)	791 (15.7%)	
> = 4	5,470(91.2%)	529 (8.8%)	
Religion			
Muslim	20,434(79.3%)	5,342 (20.7%)	0.0000
Hindu	1,506(68.2%)	701 (31.8%)	
Others	162(71.0%)	66.2 (29.0%)	
Maternal Education			
No Education	6,516(91.6%)	594 (8.4%)	0.0000
Incomplete Primary	3,991(87.5%)	570 (12.5%)	
Completed Primary	3,718(84.5%)	681 (15.5%)	
Secondary or Higher	7,877(64.9%)	4,264 (35.1%)	
ANC from a Medically Trained Provider			
No	12,935(92.5%)	1,050 (7.5%)	0.0000
Yes	9,167(64.4%)	5,059 (35.6%)	
At least 3 ANC from any Provider			
No	16,106(87.9%)	2,217 (12.1%)	0.0000
Yes	5,996(60.6%)	3,892 (39.3%)	
Watch TV Daily			
No	16,084(86.7%)	2,465 (13.3%)	0.0000
Yes	6,018(62.3%)	3,644 (37.7%)	
Read Newspaper at least weekly			
No	20,618(81.4%)	4,707 (18.6%)	0.0000
Yes	1,484(51.4%)	1,402 (48.6%)	
Complication			0.0000
Severe headache and blurred vision	4,415 (81.7%)	991 (18.3%)	
Convulsion/fits	199(68.6%)	91(31.4%)	
High Blood Pressure	221(52.3%)	202(47.7%)	
Severe heavy bleeding	154(59.6%)	104(40.4%)	
Leaking membrane	751(51.8%)	700(48.2%)	
Oedema	2,298(75.4%)	749(24.6%)	
None of the above	14,056(81.2%)	3,260(18.8%)	
Wealth Index			0.0000
Poorest	5,267 (92.5%)	426(7.5%)	
Second	5,055 (89.0%)	623(11.0%)	
Middle	4,563(82.1%)	993(17.9%)	
Fourth	4,186(74.0%)	1,469(26.0%)	
Richest	3,031(53.8%)	2,598(46.2%)	

Table 2 showed the percentage of women using health facility delivery by community level factors. Urban women were more likely to deliver in a health facility compared to rural women. Women residing in communities with a higher concentration of educated mothers, affluent households, women who have access to media and women who reported to use ANC were also more likely to deliver in a health facility.

**Table 2:** Descriptive analysis of community characteristics according to place of delivery.

	Health facility Delivery		
	No	Yes	P value
	N (%)	N (%)	
Place of Residence			
Urban	4,340 (64.8%)	2,353 (35.1%)	0.0000
Rural	17,762 (82.5%)	3,756 (17.5%)	
Region			
Barisal	1,416 (84.9%)	253 (15.1%)	0.0000
Chittagong	4,948 (80.8%)	1,172 (19.2%)	
Dhaka	7,137 (75.9%)	2,266 (24.1%)	
Khulna	2,011 (71.4%)	806 (28.6%)	
Rajshahi	4,976 (79.0%)	1,321 (21.0%)	
Sylhet	1,615 (84.7%)	291 (15.3%)	
Community exposure to newspaper			
No	22,017 (78.9%)	5,882 (21.1%)	0.0000
Yes	85 (27.1%)	227 (72.9%)	
Community exposure to TV			
No	17,771 (84.0%)	3,369 (16.0%)	0.0000
Yes	4,332 (61.3%)	2,740 (38.7%)	
Community education concentration			
Low	17,232 (82.%3)	3,709 (17.7%)	0.0000
High	4,870 (67.0%)	2,400 (33.0%)	
Community wealth concentration			
Low	16,161 (84.1%)	3,052 (15.9%)	0.0000
High	5,941 (66.0%)	3,057 (34.0%)	
Community ANC utilization			
Low	18,008 (83.4%)	3,578 (16.6%)	0.0000
High	4,094 (61.8%)	2,531 (38.2%)	
Community ANC utilization (Medically Trained Provider)			
Low	13,202 (87.6%)	1,870 (12.4%)	0.0000
High	8,900 (67.7%)	4,239 (32.3%)	

### Measures of variation (Random-effects)

We first presented an empty, intercept-only model to assess if our data justify the decision to evaluate random effects at the cluster level. As shown in Table 3, Model 1 (the empty model), there was a significant variation in the odds of delivery in a health facility across the clusters or communities (variance = 1.315 95% CI 1.192, 1.452 p—.001).

**Table 3:** Community level clustering in use of health facility delivery services.

	Model I *			Model II **			Model III ***		
	Estimate	95% CI		Estimate	95% CI		Estimate	95% CI	
Community variance (SE)	1.31	1.19	1.45	.63	.55	.73	.44	.37	.53
ICC (%)	.286	.266	.306	.162	.143	.182	.119	.102	.138

* Model I: Empty model

** Model II: Individual factors only

*** Model III: All factors (individuals and community)

The intra-class (ICC) correlation in the empty model for health facility delivery is 0.286 (95% CI 0.266, 0.306). The ICC indicates a considerable between cluster heterogeneity. A little less than one-third of the total variance in health facility delivery was attributable to the differences across the cluster or community-level factors (ICC). The variations across clusters remained statistically significant, even after controlling for all factors in the full model.

### Measures of association (Fixed-effects)

#### Individual-level factors

We then presented the full model that assessed the effect of individual, household and community level factors in the use of health facility delivery services in Table 4. The age of the women showed a significant association with the use of health facility delivery service. Relative to very young women (<20 years), women of other age groups were more likely to deliver in a health facility. Women tended to give birth at a health facility if they were educated especially with the secondary or higher level of education. Muslim women were less likely to report delivering in a health facility. The odds of delivery in a health facility decreased with increasing parity. The use of prenatal health services emerged as a strong predictor of health facility delivery. Women who had at least one ANC from a medically trained provider or had at least three ANC from any provider are more likely to use the health facility for delivery. The experience of complications during pregnancy or childbirth had increased the odds of health facility delivery. Maternal access to electronic media increased the odds of using health facility. The socioeconomic condition of the women was positively associated with the use of health facility delivery services with women in the highest wealth quintile having a 3-fold increase in the odds of delivery in a health facility compared to those in the lowest wealth quintile (OR 3.15, 95% CI 2.72–3.64). Women from urban areas were more likely to deliver in a health facility compared to women who reside in a rural community (OR 1.48, 95% CI 1.34 to 1.63).

**Table 4:** Multilevel logistic regression analysis of individual, household and community level factors associated with health facility delivery.

	Model II	Model III
	AOR (95% CI)	AOR (95% CI)
Maternal Age		
<20	1	1
20–34	1.27 (1.14, 1.42)	1.26 (1.13, 1.41)
35+	1.73 (1.47, 2.03)	1.64 (1.38, 1.95)
Parity		
1	1	1
2	2.67 (2.35, 3.03)	2.55 (2.23, 2.92)
3	1.69 (1.50, 1.90)	1.61 (1.42, 1.82)
> = 4	1.34 (1.19, 1.52)	1.30 (1.14, 1.48)
Religion		
Hindu	1	1
Muslim	0.58 (0.40, 0.83)	0.43 (0.30, 0.63)
Others*	0.89 (0.61, 1.30)	0.66 (0.45, 0.98)
Maternal Education		
No Education	1	1
Incomplete Primary	1.01 (0.89, 1.14)	1.01 (0.89, 1.15)
Completed Primary	1.01 (0.89, 1.15)	1.03 (0.90, 1.18)
Secondary or Higher	1.42 (1.26, 1.59)	1.43 (1.27, 1.61)
ANC from a Medically Trained Provider		
No	1	1
Yes	2.77 (2.56, 3.00)	2.59 (2.38, 2.83)
At least 3 ANC from any provider		
No	1	1
Yes	2.05 (1.91, 2.20)	1.91 (1.77, 2.06)
Watch TV daily		
No	1	1
Yes	1.35 (1.24, 1.45)	1.13 (1.04, 1.23)
Read Newspaper at least weekly		
No	1	1
Yes	1.40 (1.28, 1.53)	1.40 (1.27, 1.54)
Complication		
Headache & blur vision	1.22 (1.12, 1.33)	1.38 (1.26, 1.52)
Convulsion/fits	2.01 (1.52, 2.67)	2.20 (1.63, 2.98)
High Blood Pressure	3.15 (2.57, 3.85)	3.35 (2.70, 4.16)
Severe heavy bleeding	2.58 (1.91, 3.49)	2.80 (2.05, 3.83)
Leaking membrane	3.70 (3.26, 4.21)	3.89 (3.41, 4.44)
Oedema	1.19 (1.08, 1.31)	1.24 (1.12, 1.38)
None of the above	1	1
Wealth Index		
Poorest	1	1
Second	1.10 (0.97, 1.25)	1.13 (0.99, 1.30)
Middle	1.45 (1.28, 1.64)	1.53 (1.34, 1.74)
Fourth	1.70 (1.51, 1.93)	1.79 (1.57, 2.04)
Richest	2.75 (2.41, 3.15)	3.15 (2.72, 3.65)
Place of Residence		
Urban		1.48 (1.35, 1.64)
Rural		1
Region		
Sylhet		
Barisal		1.16 (0.97, 1.39)
Chittagong		0.76 (0.65, 0.88)
Dhaka		1.26 (1.08, 1.46)
Khulna		1.78 (1.51, 2.11)
Rajshahi		1.51 (1.28, 1.77)
Community exposure to newspaper		
No		1
Yes		1.64 (1.21, 2.23)
Community exposure to TV		
No		1
Yes		1.20 (1.07, 1.34)
Community education concentration		
Low		1
High		1.12 (1.02, 1.23)
Community wealth concentration		
Low		1
High		1.15 (1.03, 1.28)
Community ANC utilization (At least 3 ANC)		
Low		1
High		1.11 (1.00, 1.23)
Community ANC utilization (Medically Trained Provider)		
Low		1
High		1.25 (1.14, 1.39)

* Other religion included Buddhism and Christianity

** Model II: Individual factors only

*** Model III: All factors (individuals and community)

#### Community level factors

We found concentration of affluent households (OR 1.15, 95% CI 1.03 to 1.28); educated women (OR 1.12, 95% CI 1.02 to 1.23); use of ANC (OR 1.25, 95% CI 1.13 to 1.39); access to electronic media (OR 1.20, 95% CI 1.07 to 1.34) in a community is strongly associated with health facility delivery. The geographic region also showed a strong association with health facility delivery services. Adjusting or all other factors in the model we found the odds of using health facility delivery services were higher in Khulna, Rajshahi and Dhaka and lower in Chittagong and Barisal.

## Discussion

We found along with individual factors, community factors also have a significant influence on the use of health facility delivery services in Bangladesh. It thus confirms the findings of a previous study and reiterate the importance of community factors with respect to use of health facility delivery services[20].

Similar to other studies we found a high concentration of wealthier households in a community positively influences health facility delivery in that community[14, 21]. It is possible in communities where there is a high concentration of wealthier households, health facility delivery practice may become a norm, that other women from poorer households may follow[21]. It is also possible that in communities where there is a high concentration of wealth, health facilities function better and provide quality services, which in turn, can have a positive influence on the overall health service utilisation in the community. Our findings suggest women socioeconomic condition has a strong positive influence on health facility delivery[11, 13, 22]. These findings indicated delivering at a health health facility is influenced by the economic resources available to an individual.

Education both at individual and community level exerts a positive influence on the overall health service utilisation within the community. Several studies in Bangladesh and elsewhere have reported a strong association between women’s education and use of health facility delivery and other maternal health services [23–27]. Formal education can influence the use of health facility delivery in multiple pathways. Reproductive health education can improve knowledge and reduce reproductive health problems among adolescents in developing countries[28]. Living in a neighborhood with an educated majority expose the mother to women who are more capable of deciding on appropriate care seeking[29]. An educated woman can better catch health messages delivered through newspapers, billboards, and other media. Overall, formal education challenges traditional beliefs about health and health-seeking and transforms women’s attitudes towards safe delivery[20].

Exposure to media was also a significant predictor of health facility delivery services. Several studies have reported the effectiveness of media in influencing health service utilisation including health facility delivery[30, 31]. Higher concentration of media exposures in the community also plays an influential role in overall health service utilisation of that community. Increased media exposure might help to increase discussion of maternal issues within the community. This finding is similar to a study in Nigeria which reported the mothers residing in communities with a higher proportion of exposure to electronic and print media had higher odds of using health facility delivery services [32].

The findings that women who had more contact with antenatal care service have higher odds of using health facility delivery services might be an indication that such women are better informed about the importance of safe delivery from the counselling during antenatal care attendance. The finding is similar to the results of previous studies done in other countries [23, 24, 33, 34]. Community ANC service utilisation is also a strong predictor of health facility delivery. Higher community utilisation of ANC services indicates availability or better access to health facilities in these communities. Also, women attending antenatal care service are likely to be better informed about the danger of home delivery and could motivate their neighbours who did not participate in antenatal care service.

Place of residence and region were found to be significant predictors of health facility delivery. The result is consistent with other studies elsewhere [32, 35–37]. The difference in service utilisation among the urban and rural community as well as the different geographic region could be due to health service availability, quality of health services as well as access to health facilities. Our findings indicate that the likelihood of health facility delivery is higher among older women[20, 38] as well as multiparous women[13, 39, 40]. Earlier studies suggested older women are better aware of availability and accessibility of such services [20, 38] while multiparous woman develop confidence about childbirth from the experience and knowledge acquired from their earlier delivery[12, 26, 39]. Finally, similar to the findings of another study in Bangladesh we found Muslim women to have less probability of using health facility delivery services than women from other religions [26]. Muslim women may use fewer services at a health facility due to their conservative behaviour. After adjusting for individual and community variables, we found that there was still unexplained variance. Some of this variance might be explained by other potentially important health system level variables which we did not have data (e.g., quality of health services, availability of service provider, distance to the nearest health health facility).

### Limitations

There are few limitations in this study. We used cluster—the primary sampling unit of our research as our definition of a community. However, a cluster has an arbitrary boundary and may not represent an actual community. We did not include some known predictors of health facility service utilisation because the data were not available in the survey examined. These included the availability of health services in the community or distance to the nearest health facility. Also, we could not include husband education status or decision-making power of the women.

### Conclusion

Our findings provide valuable information to the policymakers that can be used when planning interventions to promote health facility delivery in Bangladesh. In addition to the individual attributes of women that influenced the use of health facility delivery services, we also highlighted the community determinants that contributed significantly in health facility delivery service utilization. Several community level factors significantly predicted the uptake of health facility delivery care which reinforce the need for community empowerment and focus on less privileged communities. The evidence suggests the need to go beyond addressing challenges at individual levels to improve the uptake of facility delivery services. Thus, increasing the use of health facility delivery services will require strategies that target high-risk groups, which may be most effectively defined, based on contextual factors such as community poverty, community education status, community exposure to mass media and community use of other health services. The fact that women education and household wealth are important determinants for health facility delivery services reinforces the needs for addressing current disparities in women education and wealth. Promoting intersectoral actions would thus be vital in improving maternal health. Overall, action is required at all levels–level of the individual woman and her community.
